# The Synthetic Curcumin Analogue GO-Y030 Effectively Suppresses the Development of Pressure Overload-induced Heart Failure in Mice

**DOI:** 10.1038/s41598-020-64207-w

**Published:** 2020-04-28

**Authors:** Kana Shimizu, Yoichi Sunagawa, Masafumi Funamoto, Hiroki Wakabayashi, Mai Genpei, Yusuke Miyazaki, Yasufumi Katanasaka, Nurmila Sari, Satoshi Shimizu, Ayumi Katayama, Hiroyuki Shibata, Yoshiharu Iwabuchi, Hideaki Kakeya, Hiromichi Wada, Koji Hasegawa, Tatsuya Morimoto

**Affiliations:** 1 Division of Molecular Medicine, School of Pharmaceutical Sciences, University of Shizuoka, Shizuoka, 422-8526 Japan; 2 Division of Translational Research, National Hospital Organization Kyoto Medical Center, Kyoto, 612-8555 Japan; 3 Shizuoka General Hospital, Shizuoka, 420-8527 Japan; 4 Department of Clinical Oncology, Graduate School of Medicine, Akita University, Akita, 010-8543 Japan; 5 Laboratory of Synthetic Chemistry, Department of Organic Chemistry, Tohoku University Graduate School of Pharmaceutical Sciences, Sendai, 980-8578 Japan; 6 Department of System Chemotherapy and Molecular Sciences, Division of Bioinformatics and Chemical Genomics, Graduate School of Pharmaceutical Sciences, Kyoto University, Kyoto, 606-8501 Japan

**Keywords:** Acetylation, Cardiac hypertrophy

## Abstract

Curcumin is a naturally occurring p300-histone acetyltransferase (p300-HAT) inhibitor that suppresses cardiomyocyte hypertrophy and the development of heart failure in experimental animal models. To enhance the therapeutic potential of curcumin against heart failure, we produced a series of synthetic curcumin analogues and investigated their inhibitory activity against p300-HAT. The compound with the strongest activity was further evaluated to determine its effects on cardiomyocyte hypertrophy and pressure overload-induced heart failure in mice. We synthesised five synthetic curcumin analogues and found that a compound we have named GO-Y030 most strongly inhibited p300-HAT activity. Furthermore, 1 μM GO-Y030, in a manner equivalent to 10 µM curcumin, suppressed phenylephrine-induced hypertrophic responses in cultured cardiomyocytes. In mice undergoing transverse aortic constriction surgery, administration of GO-Y030 at a mere 1% of an equivalently-effective dose of curcumin significantly attenuated cardiac hypertrophy and systolic dysfunction. In addition, this low dose of GO-Y030 almost completely blocked histone H3K9 acetylation and eliminated left ventricular fibrosis. A low dose of the synthetic curcumin analogue GO-Y030 effectively inhibits p300-HAT activity and markedly suppresses the development of heart failure in mice.

## Introduction

All types of heart disease finally lead to the development of heart failure, which is a leading cause of death worldwide. The situation has been described as an epidemic and persists despite the use of established treatment options for heart failure^1,2^. Therefore, innovative treatment strategies are urgently required. If conditions such as hypertension or myocardial infarction continue to stress the heart, it eventually leads to the failure of systolic function. Functional heart failure is closely associated with pathological cardiomyocyte hypertrophy^3,4^. Thus, controlling cardiomyocyte hypertrophy is a major target of heart failure treatment.

It is now well understood that the histone acetyltransferase (HAT) activity of the transcriptional coactivator p300 in the nucleus of cardiac myocytes plays a key role in pathological myocyte hypertrophy and heart failure^5,6^. When the heart is subjected to hypertrophic stress, neurohumoral factors regulating the renin–angiotensin system and the sympathetic nervous system are activated. These factors bind with receptors on the surface of cardiomyocytes and then activate various signalling pathways, eventually reaching the nucleus and activating p300. p300 acetylates hypertrophy-responsive transcription factors such as MEF2 and GATA4, as well as histones, thus causing pathological myocyte hypertrophy by facilitating the transcription of hypertrophy-associated genes^5,6^. *In vivo*, when transgenic mice overexpressing intact p300 in the heart undergo myocardial infarction surgery, they show significantly more progressive LV remodeling than wild-type mice undergoing the same surgery. However, when transgenic mice overexpressing mutant p300 that lacks HAT activity in the heart undergo the surgery, their degree of LV remodelling is similar to that of the wild-type mice^7^. These findings indicate that the HAT activity of p300 plays a key role in LV remodelling and systolic dysfunction, suggesting that this activity may be a target for heart failure treatment.

Curcumin ((1*E*,6*E*)-1,7-bis(4-hydroxy-3-methoxyphenyl)hepta-1,6-diene-3,5-dione) is a polyphenol derived from *Curcuma longa*. It is reported to have a variety of functions, including anticancer, antioxidant, and anti-inflammatory activities^8–10^. Additionally, Balasubramanyam *et al*. reported that curcumin inhibits p300-specific HAT activity^11^. We previously found both that curcumin suppresses cardiomyocyte hypertrophy by inhibiting the acetylation of GATA4 and histones, and that the oral administration of curcumin at a dose of 50 mg/kg prevents the development of heart failure in rat models of hypertension and myocardial infarction^12,13^. Curcumin is a natural compound and is now widely used as a dietary supplement^14,15^. However, to develop a novel drug for heart failure therapy in the clinical setting, it is necessary to synthesise novel curcumin analogues which have much stronger activity than natural curcumin.

In the present study, we examined structure–activity relationships to identify curcumin analogues that have much stronger activity against heart failure than curcumin. We investigated the inhibitory effect of five curcumin analogues on p300-HAT activity and found that GO-Y030 ((1*E*,4*E*)-1,5-bis[3,5-bis(methoxymethoxy)phenyl]-1,4-pentadiene-3-one) inhibits p300-HAT activity and the development of heart failure in mice much more strongly than does curcumin.

## Results

### The p300-HAT inhibitory activity of GO-Y030 was stronger than that of curcumin

**Figure 1:**
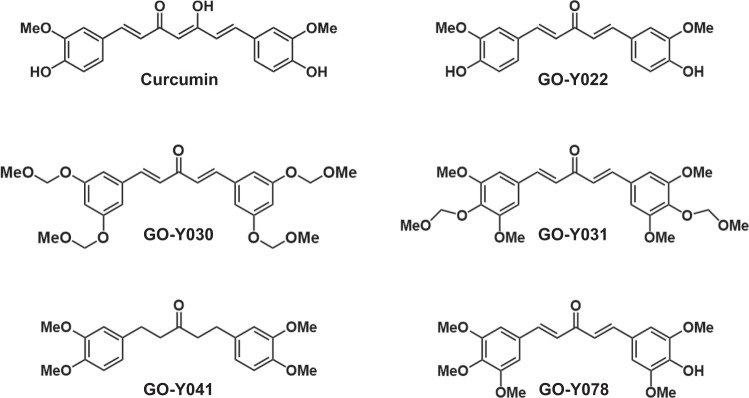
The chemical structure of curcumin and its analogues. The methoxy group is represented as MeO.

**Figure 2:**
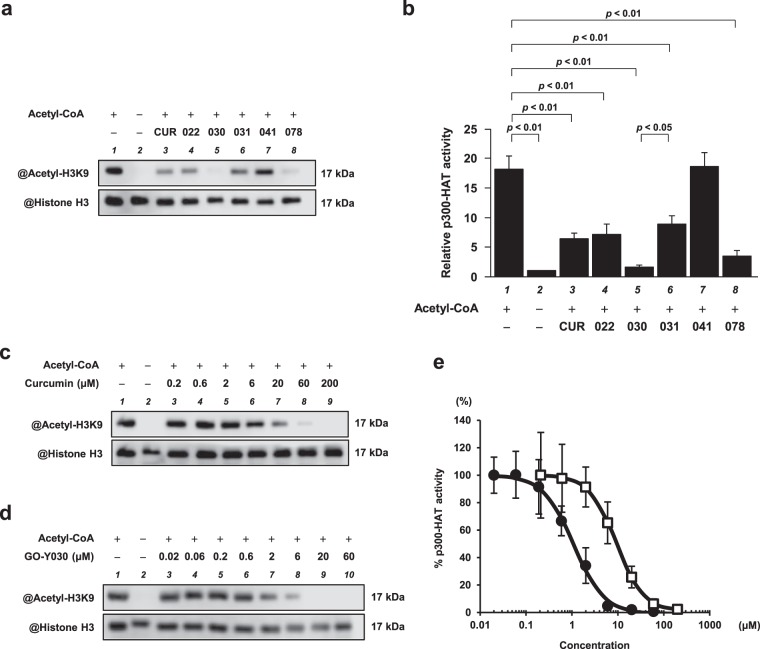
The p300-HAT inhibitory activity of GO-Y030 was stronger than that of curcumin. **(a)** All samples were subjected to western blotting with anti-acetyl-histone H3 (Lys9) antibodies and anti-histone H3 antibodies. Representative blotting images were taken using a C-DiGit Chemiluminescent Western Blot Scanner. Full-length blots are presented in Supplementary Fig. S3. **(b)** Quantification of acetylated histone H3K9 and total histone H3 levels. The quantification is presented as the mean ± SEM of three individual experiments. **(c,d)** The *in vitro* p300-HAT assay was performed with curcumin **(c)** or GO-Y030 **(d)**, and full-length blots are presented in Supplementary Figs. S4 and S5, respectively. **(e)** The concentration–response curve was obtained by plotting acetyl-histone H3K9/histone H3 vs. log [concentrations]. The IC_50_ value of GO-Y030 (●) was 1.1 µM and that of curcumin (□) was 9.4 µM. The quantified values of curcumin are presented as the mean ± SEM of three individual experiments, and GO-Y030 is presented as the mean ± SEM of five individual experiments.

To determine the structure–activity relationship of curcumin in relation to p300-HAT, an *in vitro* p300-HAT assay was performed using five curcumin analogues (Fig. 1). First, the effect of the α, β-unsaturated β-diketone moiety in the curcumin skeleton was investigated. The effect of GO-Y022, the structure of which had been changed from the β-diketone moiety in the curcumin skeleton to a monoketone moiety, was compared with the effect of curcumin. The results showed that GO-Y022 had approximately the same degree of activity as curcumin (Fig. 2a,b). In addition, to examine the contribution of the α, β-unsaturated β-diketone moiety to p300-HAT inhibition, the p300-HAT inhibitory activity of GO-Y041, which is not an α, β-unsaturated ketone, was investigated. The results revealed that GO-Y041 did not inhibit p300-HAT activity.

Next, to investigate the effect of the functional groups adducted to the aromatic rings of curcumin, GO-Y030, GO-Y031, and GO-Y078 were used in the assay. GO-Y031, which has four methoxy groups (3, 3′, 5, and 5′) and two methoxymethoxy groups (4 and 4′), inhibited p300-HAT activity to approximately the same extent as curcumin and GO-Y022. The inhibition of p300-HAT activity was enhanced by GO-Y078, in which the two methoxymethoxy groups (4 and 4′) of GO-Y031 had been further changed into a methoxy group (4) and a hydroxy group (4′). Among all the analogues, p300-HAT activity was most strongly inhibited by GO-Y030, which has four methoxymethoxy groups (3, 3′, 5, and 5′). The IC_50_ values of curcumin and GO-Y030 for p300-HAT activity were 9.4 and 1.1 µM, respectively (Fig. 2c–e).

### GO-Y030 at a dose 1/10th that of curcumin significantly suppressed PE-induced hypertrophic responses in cardiomyocytes

**Figure 3:**
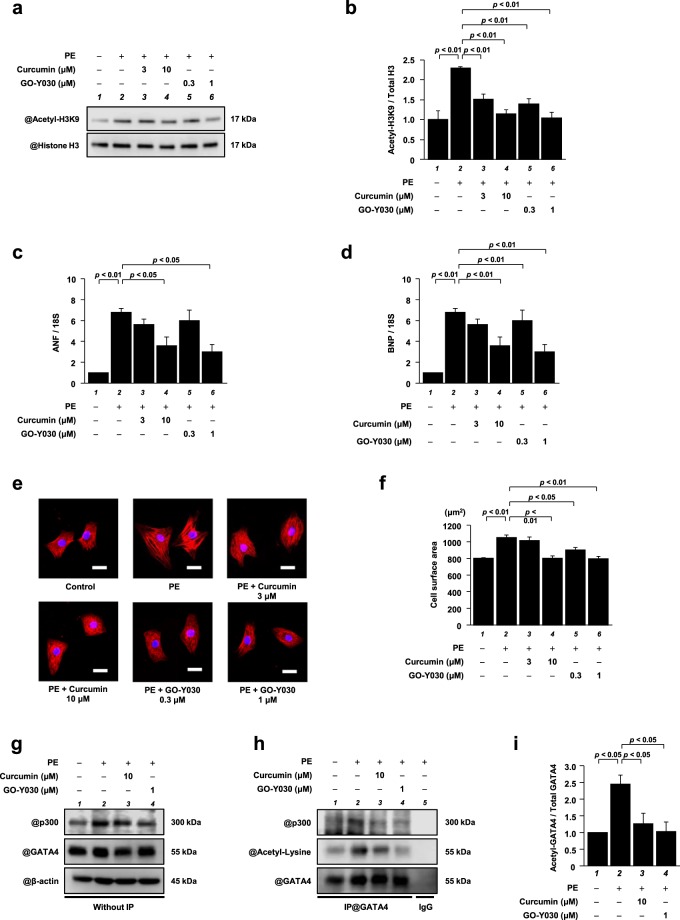
GO-Y030 at a dose 1/10th that of curcumin significantly suppressed PE-induced hypertrophic responses in cardiomyocytes. Primary cultured cardiomyocytes were treated with 3 or 10 μM curcumin, or with 0.3 or 1 μM GO-Y030 and were stimulated with 30 μM PE. **(a)** Histone fractions isolated from these cells were subjected to western blotting using anti-acetyl-histone H3 (Lys9) antibodies and anti-histone H3 antibodies. Full-length blots are presented in Supplementary Fig. S6. **(b)** The levels of acetylated histone H3K9 and total histone H3 were quantified. The data are presented as the mean ± SEM of three individual experiments. **(c,d)** Total RNA was extracted from the cells, and quantitative PCR was performed for ANF **(c)**, BNP **(d)**, and 18S. The data are presented as the mean ± SEM of three individual experiments. **(e)** Immunofluorescence staining was performed using anti-MHC antibodies and Alexa Fluor 555-conjugated anti-mouse IgG. Scale bar: 20 μm. **(f)** The surface area of these cells was measured using ImageJ software. All data are presented as the mean ± SEM of three individual experiments. **(g)** Nuclear extracts prepared from primary cultured cardiomyocytes were subjected to western blotting with anti-p300 antibodies, anti-GATA4 antibodies, and anti-β-actin antibodies. Full-length blots are presented in Supplementary Fig. S7. **(h)** The nuclear extracts were immunoprecipitated with goat anti-GATA4 polyclonal antibodies and subjected to western blotting with anti-p300 antibodies, anti-acetyl-lysine antibodies, and anti-GATA4 antibodies. The original images are presented in Supplementary Fig. S8. **(i)** The levels of acetylated GATA4 were quantified. The data are presented as the mean ± SEM of three individual experiments.

To investigate whether GO-Y030 suppresses PE-induced cardiomyocyte hypertrophy by inhibiting p300-HAT activity, primary cultured cardiomyocytes were used. The cells were stimulated with PE in the presence or absence of curcumin or GO-Y030 for 48 h. Histone fractions isolated by acid extraction from these cells were subjected to western blotting to assess acetylated histone H3K9 and total histone H3 levels. The results showed that the acetylation level of histone H3K9 was increased by PE compared with the control. The increase was significantly suppressed by low doses of GO-Y030 (0.3, 1 μM), whereas higher doses of curcumin (3, 10 μM) were required to have the same effect (Fig. 3a,b).

Next, to investigate the effect of GO-Y030 on the transcriptional activity of hypertrophic response genes, a quantified PCR analysis of ANF and BNP mRNA levels was performed. The analysis revealed that 1 µM GO-Y030 and 10 µM curcumin significantly suppressed the PE-induced increase in the mRNA levels of ANF and BNP (Fig. 3c,d). In addition, immunofluorescence staining with anti-MHC antibodies revealed that both 0.3 and 1 μM GO-Y030 significantly suppressed the PE-induced increase in the surface area of the cells, whereas 10 µM curcumin was required to have the same effect (Fig. 3e,f). These results suggest that GO-Y030 suppressed PE-induced hypertrophic responses in cardiomyocytes more strongly than curcumin.

Immunoprecipitation followed by western blotting was then performed to investigate whether GO-Y030 suppresses cardiomyocyte hypertrophy by inhibiting the p300/GATA4 pathway, as curcumin does. Nuclear protein was extracted from the cardiomyocytes, and immunoprecipitation using anti-p300 antibodies and subsequent western blotting were performed. The results showed that the PE-induced increase in the interaction between p300 and GATA4 was suppressed by 1 µM GO-Y030 to the same extent as by 10 µM curcumin (Fig. 3g). Furthermore, similar to the results of the histone acetylation, 10 µM curcumin and 1 µM GO-Y030 significantly suppressed PE-induced GATA4 acetylation (Fig. 3h,i).

### GO-Y030 at a dose 1/100th that of curcumin significantly suppressed TAC-induced cardiac hypertrophy and systolic dysfunction *in vivo*

**Figure 4:**
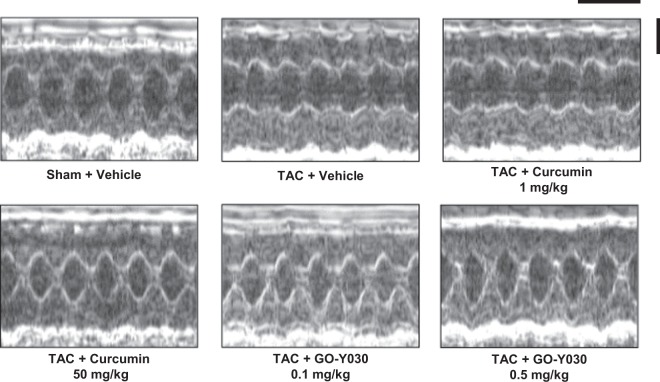
GO-Y030 at a dose 1/100th that of curcumin significantly suppressed TAC-induced cardiac hypertrophy and systolic dysfunction *in vivo*. Six weeks after the operation, cardiac function was assessed by echocardiography. Representative images of echocardiography at 6 weeks after TAC or sham operation. Horizontal bar: 200 ms. Vertical bar: 2 mm.

Because GO-Y030 strongly suppressed cardiomyocyte hypertrophy, it is likely that it also prevents pathological cardiac hypertrophy and the development of heart failure. To test this hypothesis, C57BL/6 J male mice were subjected to TAC (or to a sham operation as a control). The TAC mice were then randomly assigned to daily oral treatment with the vehicle, 1 or 50 mg/kg of curcumin, or 0.1 or 0.5 mg/kg of GO-Y030. Cardiac function was assessed by echocardiography 6 weeks after the operation. The results indicated that LVPWT, IVSd, LVIDs, and LVMI, which are parameters of cardiac hypertrophy, were increased by TAC, and that 50 mg/kg of curcumin significantly suppressed these changes. Surprisingly, 0.5 mg/kg of GO-Y030 at a dose 1/100th that of curcumin also suppressed the changes. GO-Y030 at a dose 1/100th that of curcumin also prevented the TAC-induced decrease in FS, which is a parameter of cardiac function, to the same extent as 50 mg/kg of curcumin (Fig. 4 and Table 1).

**Table 1:** Echocardiographic parameters of sham and TAC mice.

Parameter	Sham			TAC		
	Vehicle	Vehicle	CUR 1 mg/kg	CUR 50 mg/kg	GO-Y030 0.1 mg/kg	GO-Y030 0.5 mg/kg
LVPWd (mm)	1.05 ± 0.12	1.73 ± 0.17**	1.69 ± 0.09	1.43 ± 0.05^##^	1.62 ± 0.11	1.31 ± 0.09^##††^
IVSd (mm)	1.24 ± 0.11	1.63 ± 0.12**	1.62 ± 0.15	1.39 ± 0.11^#^	1.53 ± 0.09	1.38 ± 0.09^##†^
LVIDd (mm)	2.67 ± 0.26	2.83 ± 0.42	2.72 ± 0.31	2.66 ± 0.21	2.71 ± 0.22	2.67 ± 0.25
LVIDs (mm)	1.20 ± 0.27	1.81 ± 0.23**	1.71 ± 0.27	1.30 ± 0.21^##^	1.50 ± 0.36	1.27 ± 0.26^##^
LVMI (g/mg)	4.1 ± 0.6	8.3 ± 1.5**	8.0 ± 1.0	6.09 ± 0.8^##^	7.3 ± 0.6	5.6 ± 0.9^##^
FS (%)	55.6 ± 5.3	38.5 ± 7.8**	39.2 ± 4.9	52.0 ± 5.1^##^	46.1 ± 8.5	53.8 ± 6.1^##††^

*p < 0.05, **p < 0.01 vs. Sham + vehicle group.

^#^p < 0.05, ^##^p < 0.01 vs. TAC + vehicle group.

^†^p < 0.05, ^††^p < 0.01 vs. TAC + CUR 1 mg/kg group.

The values shown are the mean ± SEM for 6–7 mice from each of the sham and TAC groups.

Abbreviations: LVPWd, left ventricular posterior wall thickness; IVSd, interventricular septum thickness at end-diastole; LVIDd, left ventricular internal diameter end-diastole; LVIDs, left ventricular internal diameter end-systole; LVMI, left ventricular mass index; FS, fractional shortening

### GO-Y030 at a dose 1/100th that of curcumin significantly suppressed TAC-induced hypertrophic responses in mouse heart

**Figure 5:**
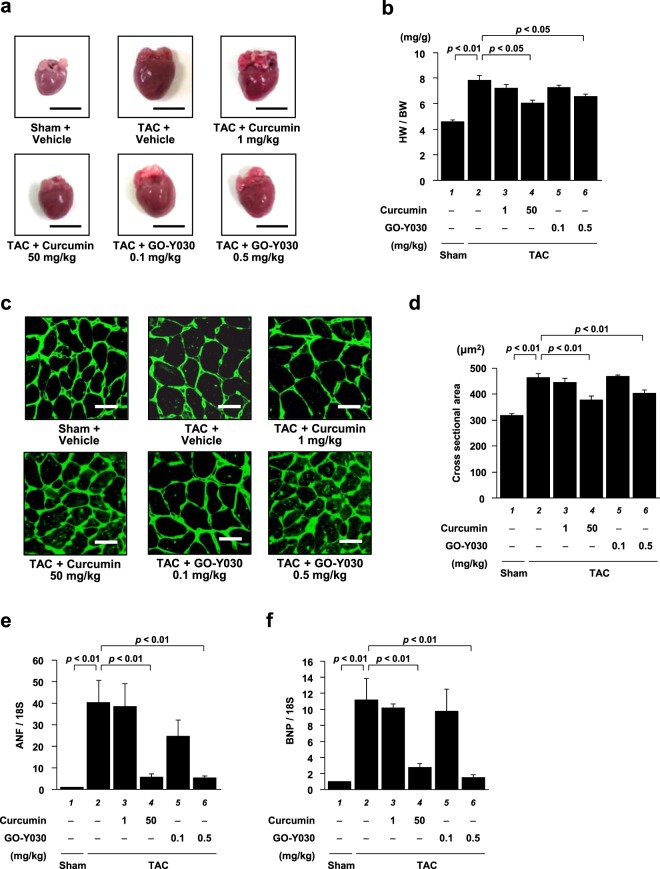
GO-Y030 at a dose 1/100th that of curcumin significantly suppressed TAC-induced hypertrophic responses in mouse heart. **(a)** The hearts were isolated from the sham and TAC groups 6 weeks after the operation. Scale bar: 5 mm. **(b)** The TAC-induced increase in HW/BW was significantly suppressed by 0.5 mg/kg of GO-Y030. The data are presented as the mean ± SEM of seven individual experiments. **(c)** Representative photographs of WGA-stained sections of LV myocardium from the sham and TAC mice. Magnification: ×400. Scale bar: 20 μm. **(d)** The cardiomyocyte cross-sectional area was measured for at least 50 cells in six to seven mice of each group. **(e,f)** Quantitative PCR analyses revealed that the mRNA levels of ANF **(e)** and BNP **(f)** were significantly suppressed by 0.5 mg/kg of GO-Y030 and 50 mg/kg of curcumin. The data are presented as the mean ± SEM of five individual experiments.

After the echocardiography assessment, the hearts were isolated (Fig. 5a) and investigated for the effect of GO-Y030 on hypertrophic responses. The heart weight and body weight of all the mice were measured, and the ratio of heart weight to body weight (HW/BW) was calculated. The results showed that 0.5 mg/kg of GO-Y030 prevented a TAC-induced increase in the HW/BW ratio to the same extent as 50 mg/kg of curcumin (Fig. 5b). Next, a histological analysis was performed by staining the heart tissues with WGA, followed by measuring the cross-sectional areas. The results showed that the two parameters were significantly increased by TAC operation and were significantly suppressed by 50 mg/kg of curcumin and 0.5 mg/kg of GO-Y030 (Fig. 5c,d). Finally, the mRNA levels of ANF and BNP in the LV were investigated by quantitative PCR analysis. As shown in Fig. 5e,f, the mRNA levels of these hypertrophic genes were significantly increased by TAC. Curcumin of 50 mg/kg significantly suppressed these increases; however, 0.5 mg/kg of GO-Y030 also suppressed them to the same extent as the 50 mg/kg of curcumin.

### GO-Y030 at a dose 1/100th that of curcumin significantly suppressed TAC-induced cardiac fibrosis

**Figure 6:**
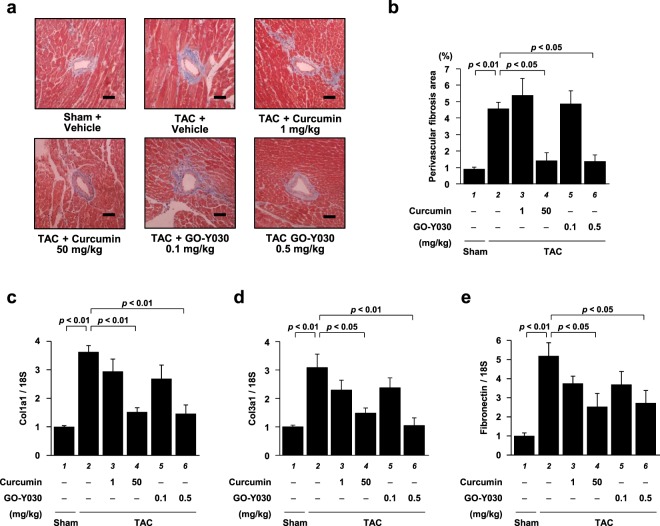
GO-Y030 at a dose 1/100th that of curcumin significantly alleviated TAC-induced cardiac inflammation and fibrosis. **(a)** Representative photographs of the MT-stained perivascular fibrosis area of LV myocardium in the sham and TAC mice. Magnification: ×200. Scale bar: 50 μm. **(b)** The area of perivascular fibrosis in the left ventricle was measured for at least three intramyocardial coronary arteries in each animal. **(c–e)** The mRNA levels of Collagen1a1 (Col1a1) **(c)**, Collagen3a1 (Col3a1) **(d)**, and Fibronectin **(e)** were significantly suppressed by GO-Y030/curcumin. The data are presented as the mean ± SEM of four to five individual experiments.

To confirm that the changes in cardiac fibrosis were indeed caused by the TAC operation and then ameliorated by the curcumin and GO-Y030 treatments, heart tissues were stained with MT, and areas of perivascular fibrogenesis were measured. The results showed that fibrotic areas were significantly increased by TAC operation and that this change was suppressed by 50 mg/kg of curcumin and 0.5 mg/kg of GO-Y030 (Fig. 6a,b). Next, the mRNA levels of genes associated with fibrosis in the mouse hearts were measured. Similar to the results of the histological analysis, collagen 1a1, collagen 3a1, and fibronectin levels were increased after the TAC operation, and 50 mg/kg of curcumin and 0.5 mg/kg of GO-Y030 significantly suppressed these increases (Fig. 6c–e).

### GO-Y030 at a dose 1/100th that of curcumin significantly suppressed TAC-induced increases in histone acetylation

**Figure 7:**
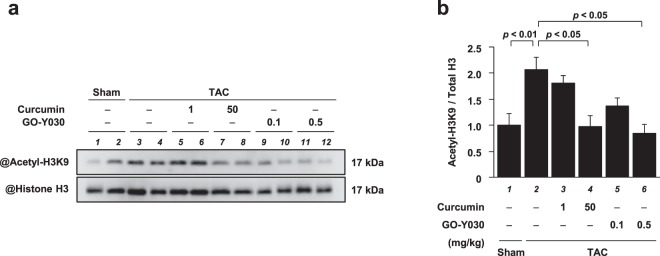
GO-Y030 at a dose 1/100th that of curcumin significantly suppressed TAC-induced increases in histone acetylation. **(a,b) (a)** Histone fractions from the mouse hearts were subjected to western blotting to assess acetylated histone H3K9 and total histone H3 levels. Full-length blots are presented in Supplementary Fig. S9. **(b)** The levels of acetylated histone H3K9 and total histone H3 were quantified. All data are presented as the mean ± SEM (n = 4).

To determine whether a low dose of GO-Y030 can suppress TAC-induced histone acetylation, western blotting was performed using histone fractions from the hearts. The acetylation of histone H3K9 was also enhanced by TAC; however, 0.5 mg/kg of GO-Y030 suppressed the increase in this acetylation to the same extent as 50 mg/kg of curcumin (Fig. 7a,b). This suggests that the improvement in heart failure induced by GO-Y030 is attributable to its strong inhibition of p300-HAT activity.

### GO-Y030 toxicity was not observed in the TAC mice at a concentration of 0.5 mg/kg

Finally, the toxicity of GO-Y030 in mice was investigated. Blood was collected from the mice 6 weeks after the TAC operation. Markers of liver function, including alanine transferase, aspartate transaminase, and total bilirubin, and markers of renal function, including creatinine and blood urea nitrogen, in the blood were tested. There were no differences in any parameters among the three groups: TAC + vehicle, curcumin, or GO-Y030 (Supplementary Fig. S1a–e). Next, the weights of the liver and kidney were corrected for body weight. No significant differences in liver weight to body weight or kidney weight to body weight were found among the four groups (Supplementary Fig. S1f,g). In addition, the liver and kidney tissues were stained with HE and periodic acid–Schiff staining, respectively, and were observed by microscopy. After the administration of curcumin or GO-Y030, no disruption of hepatic lobules was observed, and there were no aberrations in the glomerulus or mesangial matrix (Supplementary Fig. S2a,b).

## Discussion

This study demonstrates that GO-Y030 inhibits p300-HAT activity and PE-induced hypertrophy in cultured cardiomyocytes at 1/10th the dose of curcumin. More importantly, the oral administration of GO-Y030 ameliorated TAC-induced cardiac hypertrophy and heart failure at 1/100th the dose of curcumin. The high effectiveness of GO-Y030 at a low dose may overcome the problem of the very large dose of curcumin that would be needed in clinical settings due to its low bioavailability. This may enable the development of an effective new treatment for heart failure.

The results of the structure–activity relationship study determined by the *in vitro* p300-HAT assay revealed that GO-Y030 inhibited p300-HAT activity most strongly among all the analogues and that it had p300-HAT inhibitory activity that was approximately nine times higher than that of curcumin. There are two key structural differences between curcumin and GO-Y030 that potentially explain this difference in their degree of p300-HAT inhibitory activity: the differences in their ketone structures and the differences in the structure of their functional groups. Evidence from this and other studies suggests that the decisive difference is in their functional groups. In terms of ketone structure, both curcumin and GO-Y030 are α, β-unsaturated ketones. Both have been reported to act as Michael acceptors; the α, β-unsaturated ketone structure of curcumin has been reported to bind covalently to p300, and it appears very likely that that of GO-Y030 does as well^16–20^. However, there is a key difference in the structures of the two ketones: curcumin is an α, β-unsaturated β-diketone, whereas GO-Y030 is an α, β-unsaturated monoketone. The present study found that the analogue GO-Y022, which is also a monoketone, had the same degree of p300-HAT inhibitory activity as curcumin. This finding suggests that there is no difference in binding affinity in the Michael reaction between the α, β-unsaturated β-diketone structure of curcumin and the α, β-unsaturated monoketone structure of the analogues. This view is additionally supported by the fact that the analogue GO-Y041, which does not have an unsaturated ketone structure, did not inhibit p300-HAT activity, most likely because it did not bind to p300 due to its lack of a Michael acceptor.

Unlike the differences in ketone structures, the differences in the position and type of the functional groups attached to the aromatic rings appear to strongly affect the degree of p300-HAT inhibitory activity. GO-Y030, which has two methoxymethoxy groups (3 and 5) on each aromatic ring, had much greater inhibitory activity than GO-Y022, which has a methoxy group (3) and a hydroxyl group (4) on each ring. Moreover, the fact that GO-Y078, which has three methoxy groups (3, 4 and 5) on one ring and two methoxy groups (3′ and 5′) and a hydroxyl group (4′) on another, inhibited p300-HAT activity more strongly than GO-Y031, which has two methoxy groups (3 and 5) and a methoxymethoxy group (4) on each ring. This finding suggests that the addition of a large functional group, such as the methoxymethoxy group at position 4, reduces binding affinity to p300. It has previously been reported that the functional groups on the aromatic rings of curcumin are involved in hydrogen bonding to p300^19^. Furthermore, we previously reported that the methoxy groups at position 3 did not affect p300-HAT inhibitory activity^21^. Taken together, these findings suggest that the addition of a methoxy group or a methoxymethoxy group at position 5 is important for binding affinity to p300 via the hydrogen bond. Thus, the greater p300-HAT inhibitory activity of GO-Y030 compared with that of curcumin can be attributed to the differences in the functional groups of the two compounds, rather than to the differences in their ketone structures.

Our experiment with cultured cardiomyocytes revealed that curcumin inhibited cardiomyocyte hypertrophy and the acetylation of histones at a concentration of 10 μM, whereas GO-Y030 did so at 1 μM, i.e., 1/10th the concentration of curcumin. The results of both the *in vitro* HAT assay and the cultured cardiomyocyte experiment showed that GO-Y030 had the same degree of inhibitory effect as curcumin at 1/10th the concentration; therefore, as the cell permeability of GO-Y030 and curcumin in cardiomyocytes was roughly the same, and as GO-Y030 was a more potent inhibitor of p300-HAT activity than curcumin in the cardiomyocyte nucleus, it can be concluded that GO-Y030 suppressed cultured cardiomyocyte hypertrophy at a lower concentration than curcumin.

GO-Y030, at a dose 1/100th that of curcumin, ameliorated cardiac hypertrophy and cardiac dysfunction in a pressure overload model. Additionally, it inhibited perivascular fibrosis and hypertrophy of individual cardiomyocytes, and it inhibited histone acetylation. This reveals that, relative to curcumin, GO-Y030 had a 10-fold greater effect *in vivo* than in cultured cardiomyocytes. There are several possible explanations for this difference in effect. First, it is possible that GO-Y030 acts on other cells in addition to cardiomyocytes and therefore additively suppresses the development of heart failure. Cardiac fibrosis is closely related to the progression of heart failure^22,23^, and we have found that GO-Y030 suppressed the fibrotic response at a lower concentration than curcumin in primary cultured cardiac fibroblasts (data not shown). Thus, it can be assumed that GO-Y030 had a much greater effect *in vivo* than in cultured cardiomyocytes because it affected both cardiomyocytes and cardiac fibroblasts. Second, it is possible that GO-Y030 has greater bioavailability than curcumin. It has been reported that the low bioavailability of curcumin is due to hydrophobicity, poor absorption, and rapid metabolism^24^. Differences in the structure of curcumin and GO-Y030 may affect these factors, potentially improving the bioavailability of GO-Y030 over that of curcumin. The present study also suggests that GO-Y030 is safe, finding no toxicity for the 0.5 mg/kg dose at six weeks. This confirms the results of previous studies, which found that mice had no adverse reactions to the administration of feed containing 0.1% GO-Y030 for 2 months^25,26^. Further investigation of the bioavailability and possible side effects of GO-Y030 is required to assess its potential clinical application.

The present study has also demonstrated that 1 µM GO-Y030 suppressed the activation of the p300/GATA4 signaling pathway in cardiomyocyte hypertrophy as well as 10 µM curcumin did. p300 controls gene expression by acetylating histone and transcriptional factors; it also regulates transcriptional factors by forming a transcriptional complex as a scaffold protein^27,28^. We previously reported that curcumin suppressed the development of heart failure not only by inhibiting the acetylation of histone and GATA4 but also by interrupting the formation of the p300/GATA4 complex^12^. In this study, GO-Y030 also inhibited both the acetylation of histone and GATA4 and the interaction between p300 and GATA4. These results suggest that GO-Y030 suppressed the development of heart failure via the same mechanisms as curcumin.

In summary, this study has demonstrated that the curcumin analogue GO-Y030 effectively inhibits p300-HAT activity and ameliorates TAC-induced progression of cardiac hypertrophy and heart failure at much lower doses than the natural compound curcumin. These findings suggest that GO-Y030 may have a therapeutic effectiveness in the clinical setting equal to that of curcumin at a much lower dosage. This would be a great benefit both to elderly patients who have difficulty swallowing a large volume of oral medications and to patients under fluid restriction conditions who must keep their water intake low when taking oral medications. Further studies are expected to apply this novel drug to heart failure patients in clinical settings.

## Materials and Methods

### Materials

Curcumin was purchased from Nagara Science Corporation (Gifu, Japan). The curcumin analogues GO-Y022, GO-Y030, GO-Y031, GO-Y041, and GO-Y078 were synthesised as described previously^25^. These compounds were dissolved in dimethyl sulfoxide and stored at −20 °C.

### *In vitro* p300-HAT assay

The *in vitro p300-*HAT assay was performed as described previously^21^. In brief, 5 μg of core histones from calf thymus (Worthington, USA) was incubated in HAT buffer with purified p300-HAT recombinant domain in the presence of curcumin or GO-Y030 at room temperature for 30 min. The reactions were initiated by adding acetyl-CoA to each sample and then incubating the sample for 30 min. All samples were subjected to SDS polyacrylamide gel electrophoresis (SDS-PAGE) followed by western blotting with rabbit polyclonal anti-acetyl-histone H3 (Lys9) antibodies and rabbit polyclonal anti-histone H3 antibodies (Cell Signalling Technology, USA). The 50% inhibitory concentration (IC_50_) was calculated from the concentration–response curve.

### Animal experiments

Male Sprague-Dawley rats were purchased from Japan SLC Inc. (Shizuoka, Japan). C57BL/6 J male mice were purchased from CREA Japan Inc. (Tokyo, Japan). All animal experiments complied with the guidelines on animal experiments of the University of Shizuoka and National Hospital Organization Kyoto Medical Center and were performed in accordance with protocols approved by University of Shizuoka Ethics Committee and (number 156161) and the National Hospital Organization Kyoto Medical Center Ethics Committee (number 27-26-2).

### Cell culture

Primary cultured cardiomyocytes were isolated from 1–2-day-old Sprague-Dawley rats as described previously^12,29^. The cells were pretreated with 3 or 10 μM curcumin or 0.3 or 1 μM GO-Y030. Two hours after treatment, the cells were stimulated with or without 30 μM PE for 48 h.

### Detection of histone acetylation

Histone fractions from the cultured cardiomyocytes and mouse hearts were isolated by acid extraction as described previously^12,30^. The samples were resolved by SDS-PAGE. Acetylated histone H3K9 and total histone H3 were detected by western blotting with an anti-acetyl-histone H3 (Lys9) antibody and a rabbit polyclonal anti-histone H3 antibody, respectively. Western blotting signals were visualised using a C-DiGit Chemiluminescent Western Blot Scanner (LI-COR, USA) and quantified with Image Studio LITE software (LI-COR).

### Real-time polymerase chain reaction

Real-time polymerase chain reaction was performed as described previously^12,29^. In brief, total RNA from the cardiomyocytes and LVs of the mice was extracted using TRI Reagent (Invitrogen, USA). Reverse transcription polymerase chain reaction (RT-PCR) was performed using ReverTra Ace^®^ qPCR RT Master Mix (Toyobo, Osaka, Japan). Quantitative PCR was performed with a LightCycler 96 Real-Time PCR System (Roche, Switzerland) with KOD SYBR qPCR Mix (Toyobo). Rat 18 S was used as an internal control^11,12^. Supplementary Table 1 contains a list of the gene primer sequences, including hypertrophic, fibrotic, and inflammatory response genes.

### Immunofluorescent staining and measurement of cardiomyocyte surface area

Immunofluorescent staining of the cultured cardiomyocytes was performed as described previously^12,29^. The cardiomyocytes were cultured in glass chamber slides (Nalge Nunc International, USA) and were stained with anti-myosin heavy chain (MHC) antibodies (Leica Biosystems, Germany) and Alexa Fluor 555-conjugated anti-mouse IgG (Invitrogen) using the indirect immunoperoxidase method. Hoechst 33258 (Dojinjo, Kumamoto, Japan) was used for nuclear staining. Fifty cardiomyocytes were randomly selected from each group, and the surface area of these cells was measured using ImageJ software (version l.33 u).

### Immunoprecipitation and western blotting

Immunoprecipitation and western blotting for acetylated lysine and GATA4 were performed as previously described^12,30^. For immunoprecipitation, goat anti-GATA4 polyclonal antibodies (Santa Cruz Biotechnology Inc., USA) were used. For western blotting, rabbit polyclonal antibodies against acetylated lysine (Cell Signalling), mouse anti-GATA4 polyclonal antibodies (Cell Signalling), rabbit anti-p300 polyclonal antibodies (Santa Cruz Biotechnology Inc.), and mouse anti-β-actin monoclonal antibodies (Sigma-Aldrich, USA) were used.

### Transverse aortic constriction (TAC)

C57BL/6J male mice (8 weeks old) were anaesthetised with 1.0–1.5% isoflurane and their limbs were anchored. While the mice were connected to a ventilator (0.1–0.3 mL/min, 150 times/min), the pleura was incised to the second rib, and the aortic arch was ligated using a 7–0 nylon suture ligature with a 27-gauge needle. The needle was then promptly removed, and the intercostal muscle and skin were sutured using 5–0 nylon suture ligature. A sham operation was performed with the same surgical procedure, except that the suture around the aortic arch was not tied.

### Drug treatments

One day after the operation, the 86 mice that had undergone the TAC operation were randomly assigned to five groups: vehicle (1% gum Arabic, n = 18), 1 mg/kg curcumin (n = 17), 50 mg/kg curcumin (n = 15), 0.1 mg/kg GO-Y030 (n = 18), and 0.5 mg/kg GO-Y030 (n = 18). The compounds were dissolved with 1% gum Arabic and administrated to the mice orally by gastric gavage once a day for 6 weeks. The sham mice (n = 14) were treated with the vehicle orally.

### Echocardiography

Echocardiography was performed using a 10–12 MHz probe and a Sonos 5500 Ultrasound System (Philips, The Netherlands) as described previously^12,31^. Interventricular septum thickness at end-diastole (IVSd), left ventricular internal diameter end-diastole (LVIDd), left ventricular internal diameter end-systole (LVIDs), and left ventricular posterior wall thickness (LVPWT) were obtained from M-mode recordings. Fractional shortening (FS) and LV mass were calculated as (LVIDd − LVIDs)/LVIDd × 100 (%) and 1.055 [(IVSd + LVIDd + LVPWT)^3^ − (LVIDd)^3^], respectively. LV mass index (LVMI) is represented as the ratio of LV mass to body weight.

### Histological analysis

The mice were euthanised, and their hearts were isolated and cut into two transverse slices at the mid-level of the papillary muscles. The samples were fixed with 10% formalin and embedded in paraffin. They were then stained with FITC-conjugated wheat germ agglutinin (WGA) and Masson trichrome (MT). The perivascular fibrotic area was measured as described previously^12,31,32^. The sections were deparaffinised and incubated with FITC-conjugated WGA (Sigma-Aldrich) diluted 1:100 (10 μg/mL) in 1% BSA/PBS for 60 min while being protected from light. From each group, several sections were randomly photographed with a fluorescence microscope (LSM 510 META, Zeiss, Japan), and the surface areas of 50 cardiomyocytes were measured using ImageJ software. MT-stained perivascular sections were photographed using an Eclipse 80i microscope (Nikon, Japan). The areas of perivascular fibrogenesis were measured using ImageJ software, and the resulting value divided by the total area of the photograph (12,288 pixels) was regarded as the relative vascularised fibrosis area. The entire heart was imaged with a Leica TL5000 Ergo microscope (Leica Microsystems, Japan), and the area of interstitial fibrosis was measured using ImageJ software. The results are represented as the relative fibrosis area (% of total myocardial area).

### Statistics

Values are shown as the mean ± SEM from at least three independent experiments. Statistical comparisons were performed using ANOVA with the Tukey–Kramer test. A *p* value of < 0.05 was considered statistically significant.

## Supplementary information

Supplementary materials.

## References

[1] Roger VL (2013). Epidemiology of Heart Failure. Circ Res..

[2] Bui AL, Horwich TB, Fonarow GC (2011). Epidemiology and risk profile of heart failure. Nat Rev Cardiol..

[3] Rosca MG, Tandler B, Hoppel CL (2013). Mitochondria in cardiac hypertrophy and heart failure. J Mol Cell Cardiol..

[4] Lips DJ, deWindt LJ, van Kraaij DJ, Doevendans PA (2003). Molecular determinants of myocardial hypertrophy and failure: alternative pathways for beneficial and maladaptive hypertrophy. Eur Heart J..

[5] Yanazume T (2003). Cardiac p300 is involved in myocyte growth with decompensated heart failure. Mol Cell Biol..

[6] Wei JQ (2008). Quantitative control of adaptive cardiac hypertrophy by acetyltransferase p300. Circulation..

[7] Miyamoto S (2006). Histone acetyltransferase activity of p300 is required for the promotion of left ventricular remodeling after myocardial infarction in adult mice *in vivo*. Circulation..

[8] Tsuda T (2018). Curcumin as a functional food-derived factor: degradation products, metabolites, bioactivity, and future perspectives. Food Funct..

[9] Joe B, Vijaykumar M, Lokesh BR (2004). Biological properties of curcumin-cellular and molecular mechanisms of action. Crit Rev Food Sci Nutr..

[10] Anand P (2008). Biological activities of curcumin and its analogues (Congeners) made by man and Mother Nature. Biochem Pharmacol..

[11] Balasubramanyam K (2004). Curcumin, a novel p300/CREB-binding protein-specific inhibitor of acetyltransferase, represses the acetylation of histone/nonhistone proteins and histone acetyltransferase-dependent chromatin transcription. J Boil Chem..

[12] Morimoto T (2008). The dietary compound curcumin inhibits p300 histone acetyltransferase activity and prevents heart failure in rats. J Clin Invest..

[13] Sunagawa Y (2014). Optimal Dose-Setting Study of Curcumin for Improvement of Left Ventricular Systolic Function After Myocardial Infarction in Rats. J Pharmacol Sci..

[14] Funamoto M (2016). Highly absorptive curcumin reduces serum atherosclerotic low-density lipoprotein levels in patients with mild COPD. Int J Chron Obstruct Pulmon Dis..

[15] Zeitlin P (2004). Can curcumin cure cystic fibrosis?. N Engl J Med..

[16] Marcu MG (2006). Curcumin is an inhibitor of p300 histone acetylatransferase. Med Chem..

[17] Salem M, Rohani S, Gillies ER (2014). Curcumin, a promising anti-cancer therapeutic: a review of its chemical properties, bioactivity and approaches to cancer cell delivery. RSC Adv..

[18] Ravindra KC, Narayan V, Lushington GH, Peterson BR, Prabhu KS (2012). Targeting of Histone Acetyltransferase p300 by Cyclopentenone Prostaglandin Δ12-PGJ2 through Covalent Binding to Cys1438. Chem Res Toxicol..

[19] Devipriya B, Kumaradhas P (2013). Molecular flexibility and the electrostatic moments of curcumin and its derivatives in the active site of p300: a theoretical charge density study. Chem Biol Interact..

[20] Kohyama A (2016). Reversibility of the thia-Michael reaction of cytotoxic C5-curcuminoid and structure-activity relationship of bis-thiol-adducts thereof. Org Biomol Chem..

[21] Sunagawa Y (2018). Curcumin derivative, demethoxycurcumin and bisdemethoxycurcumin, possess p300 HAT inhibitory activity and suppress hypertrophicresponses in cardiomyocyte. J Pharmacol Sci..

[22] Krenning G, Zeisberg EM, Kalluri R (2010). The origin of fibroblasts and mechanism of cardiac fibrosis. J Cell Physiol..

[23] Bacmeister L (2019). Inflammation and fibrosis in murine models of heart failure. Basic Res Cardiol..

[24] Mohanty C, Das M, Sahoo SK (2012). Emerging role of nanocarriers to increase the solubility and bioavailability of curcumin. Expert Opin Drug Deliv..

[25] Ohori H (2006). Synthesis and biological analysis of new curcumin analogues bearing an enhanced potential for the medicinal treatment of cancer. Mol Cancer Ther..

[26] Shibata H (2009). Newly synthesized curcumin analog has improved potential to prevent colorectal carcinogenesis *in vivo*. Cancer Sci..

[27] Dancy BM, Cole PA (2015). Protein lysine acetylation by p300/CBP. Chem Rev..

[28] Dyson HJ, Wright PE (2016). Role of Intrinsic Protein Disorder in the Function and Interactions of the Transcriptional Coactivators CREB-binding Protein (CBP) and p300. J Biol Chem..

[29] Suzuki H (2016). Tyrosine phosphorylation of RACK1 triggers cardiomyocyte hypertrophy by regulating the interaction between p300 and GATA4. Biochim Biophys Acta..

[30] Sunagawa Y (2010). Cyclin-dependent kinase-9 is a component of the p300/GATA4 complex required for phenylephrine-induced hypertrophy in cardiomyocytes. J Biol Chem..

[31] Sunagawa Y (2012). A novel drug delivery system of oral curcumin markedly improves efficacy of treatment for heart failure after myocardial infarction in rats. Biol Pharm Bull..

[32] Sunagawa Y (2011). A natural p300-specific histone acetyltransferase inhibitor, curcumin, in addition to angiotensin-converting enzyme inhibitor, exerts beneficial effects on left ventricular systolic function after myocardial infarction in rats. Circ J..

