# Supplementary material for: The Synthetic Curcumin Analogue GO-Y030 Effectively Suppresses the Development of Pressure Overload-induced Heart Failure in Mice
Source: Sci Rep. 2020 Apr 28;10:7172. doi: 10.1038/s41598-020-64207-w (PMC7188884; doi:10.1038/s41598-020-64207-w)
Supplement: Supplementary file 1 — Supplementary materials. [file 41598_2020_64207_MOESM1_ESM.docx]

**The Synthetic Curcumin Analogue GO-Y030 Effectively Suppresses the Development of Pressure Overload-induced Heart Failure in Mice**

KANA SHIMIZU^1, 2^ ; YOICHI SUNAGAWA^1, 2, 3^ ; MASAFUMI FUNAMOTO^1, 2^ ; HIROKI WAKABAYASHI^1^ ; MAI GENPEI^1^ ; YUSUKE MIYAZAKI^1, 2, 3^ ; YASUFUMI KATANASAKA^1, 2, 3^ ; NURMILA SARI^1^; SATOSHI SHIMIZU^1, 2^; AYUMI KATAYAMA^1^; HIROYUKI SHIBATA^4^ ; YOSHIHARU IWABUCHI^5^ ; HIDEAKI KAKEYA^6^ ; HIROMICHI WADA^2^ ; KOJI HASEGAWA^1, 2^ ; TATSUYA MORIMOTO^1, 2, 3^

^1^ Division of Molecular Medicine, School of Pharmaceutical Sciences, University of Shizuoka, Shizuoka, 422-8526, Japan

^2^ Division of Translational Research, National Hospital Organization Kyoto Medical Center, Kyoto, 612-8555, Japan

^3^ Shizuoka General Hospital, Shizuoka, 420-8527, Japan

^4^ Department of Clinical Oncology, Graduate School of Medicine, Akita University, Akita, 010-8543, Japan

^5^ Laboratory of Synthetic Chemistry, Department of Organic Chemistry, Tohoku University Graduate School of Pharmaceutical Sciences, Sendai, 980-8578, Japan

^6^ Department of System Chemotherapy and Molecular Sciences, Division of Bioinformatics and Chemical Genomics, Graduate School of Pharmaceutical Sciences, Kyoto University, Kyoto, 606-8501, Japan

*Address correspondence to: Tatsuya Morimoto, M.D., Ph.D.

Division of Molecular Medicine, School of Pharmaceutical Sciences, University of Shizuoka

TEL: 81-54-264-5763 FAX: 81-54-264-5764

e-mail: [morimoto@u-shizuoka-ken.ac.jp](mailto:morimoto@u-shizuoka-ken.ac.jp)


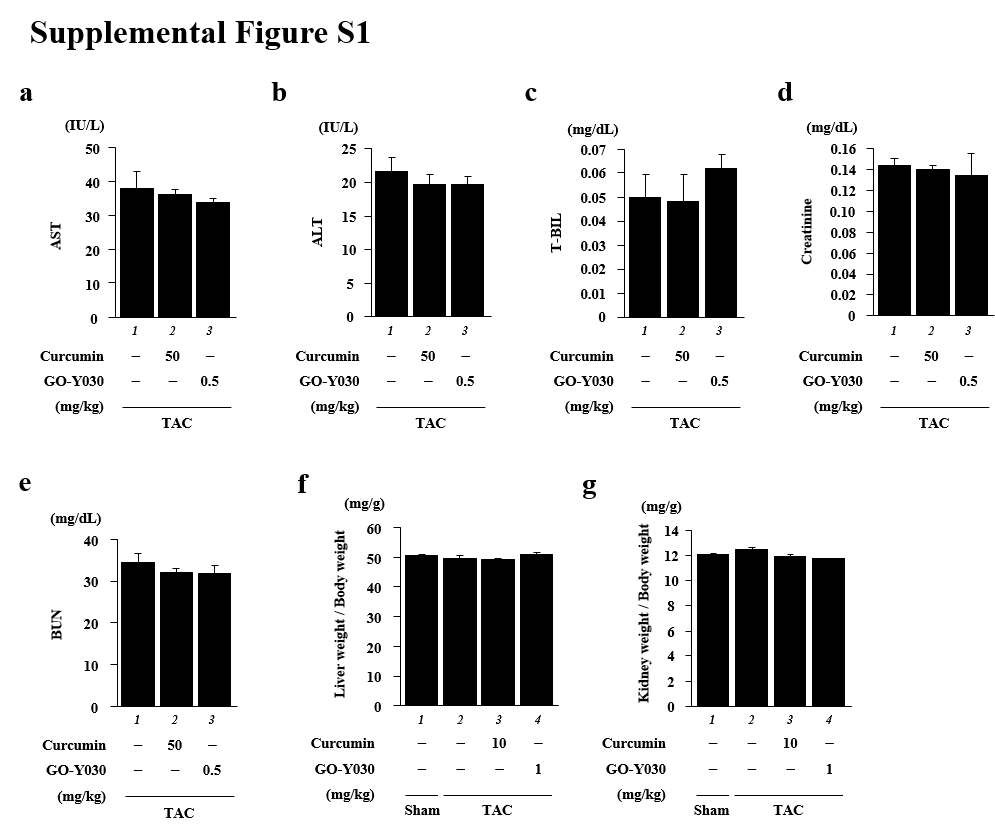


**Supplementary Fig. S1 Curcumin and GO-Y030 toxicity were not observed in liver or kidney.**

**(a-e)** Six weeks after TAC operation, blood was sampled from all mice. Serum levels of aspartate aminotransferase (AST) **(a)**, alanine aminotransferase (ALT) **(b)**, total bilirubin (T-BIL) **(c)**, creatinine **(d)**, and blood urea nitrogen **(**BUN) **(e)** were measured. There was no difference in any parameters among TAC mice treated with vehicle, curcumin, or GO-Y030. The data are presented as the mean ± SEM of five individual experiments. **(f and g)** Liver weight **(f)** and kidney weight **(g)** were corrected for body weight. The data are presented as the mean ± SEM of eight individual experiments.


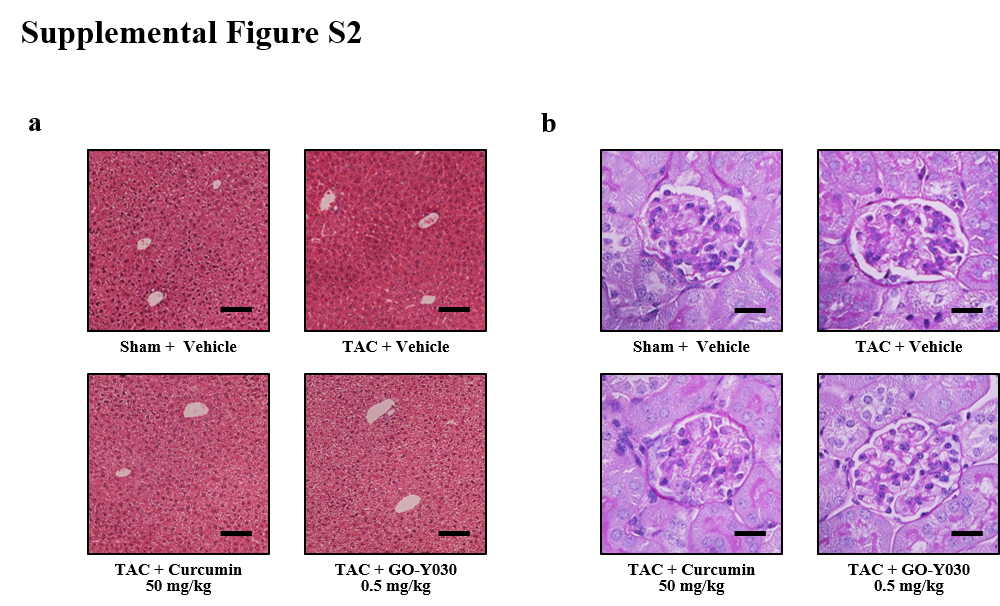


**Supplementary Fig. S2 Curcumin and GO-Y030 induced neither disruption of hepatic lobules, nor aberration of glomerulus or the mesangial matrix.**

**(a)** Representative images of HE-stained liver 6 weeks after TAC operation. Magnification: ×100. Scale bar: 100 μm. **(b)** Representative images of periodic acid-Schiff-stained kidney 6 weeks after TAC operation. Magnification: ×400. Scale bar: 20 μm.


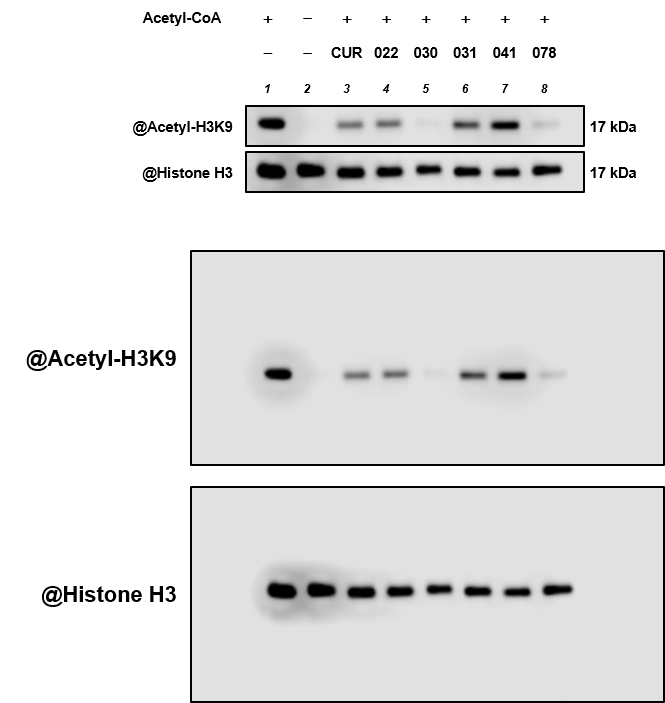


**Supplementary Fig. S3 Original western blotting images in Figure 2a**

An *in vitro* p300-HAT assay using a recombinant p300-HAT domain and histones was performed with 10 µM curcumin and its analogues: CUR, curcumin; 022, GO-Y022; 030, GO-Y030; 031, GO-Y031; 41, GO-Y041; 078, GO-Y078.


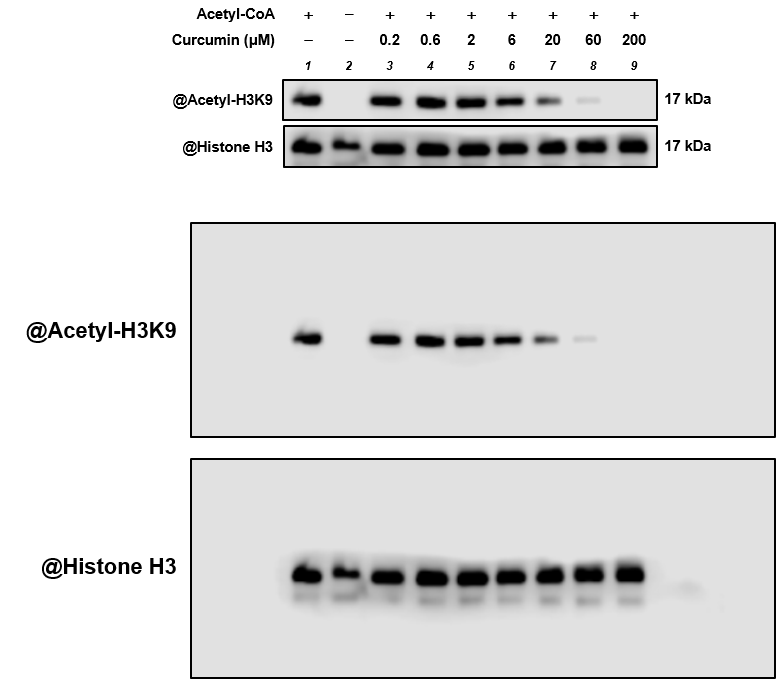
**Supplementary Fig. S4 Original western blotting images in Figure 2c**

An *in vitro* p300-HAT assay was performed with curcumin.


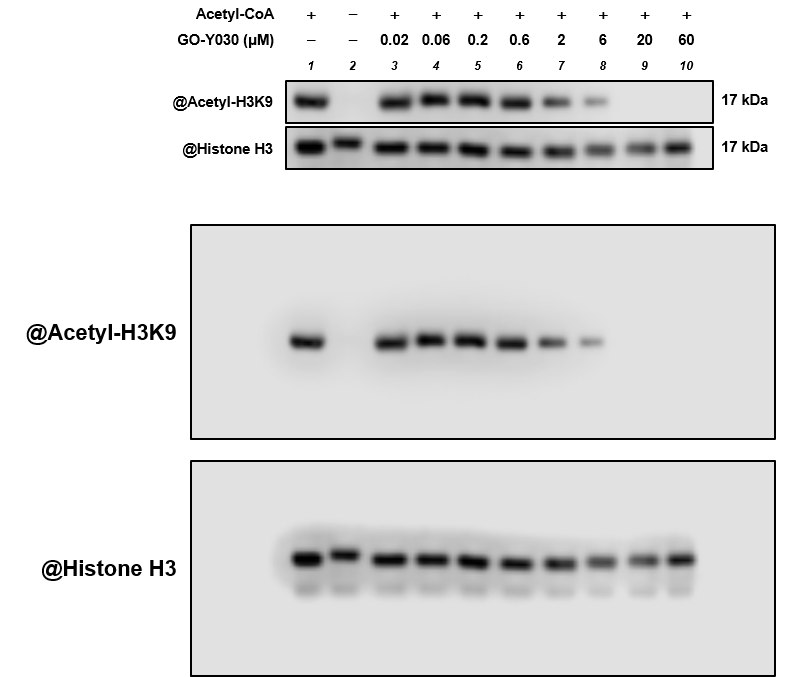


**Supplementary Fig. S5 Original western blotting images in Figure 2d**

An *in vitro* p300-HAT assay was performed with GO-Y030.


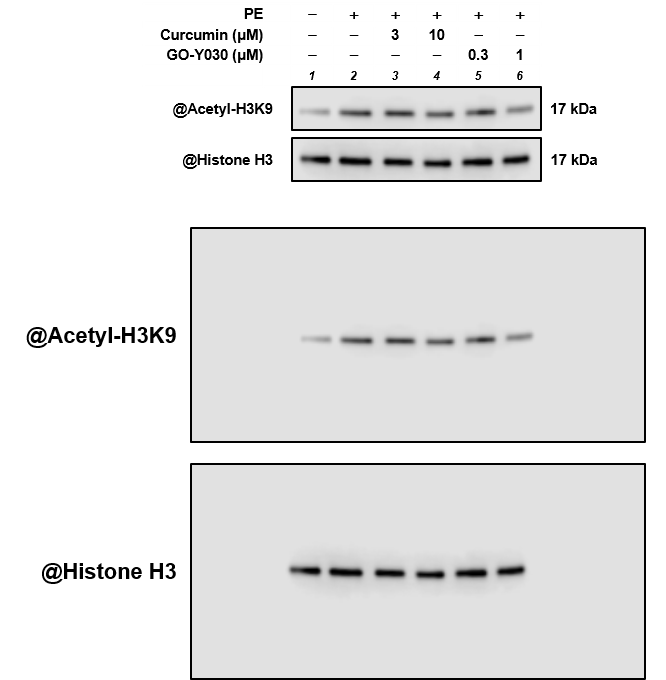


**Supplementary Fig. S6 Original western blotting images in Figure 3a**

Primary cultured cardiomyocytes were treated with 3 or 10 μM curcumin, or with 0.3 or 1 μM GO-Y030, and were then stimulated with 30 μM phenylephrine (PE). Histone fractions isolated from these cells were subjected to western blotting using anti-acetyl-histone H3 (Lys9) antibodies and anti-histone H3 antibodies.


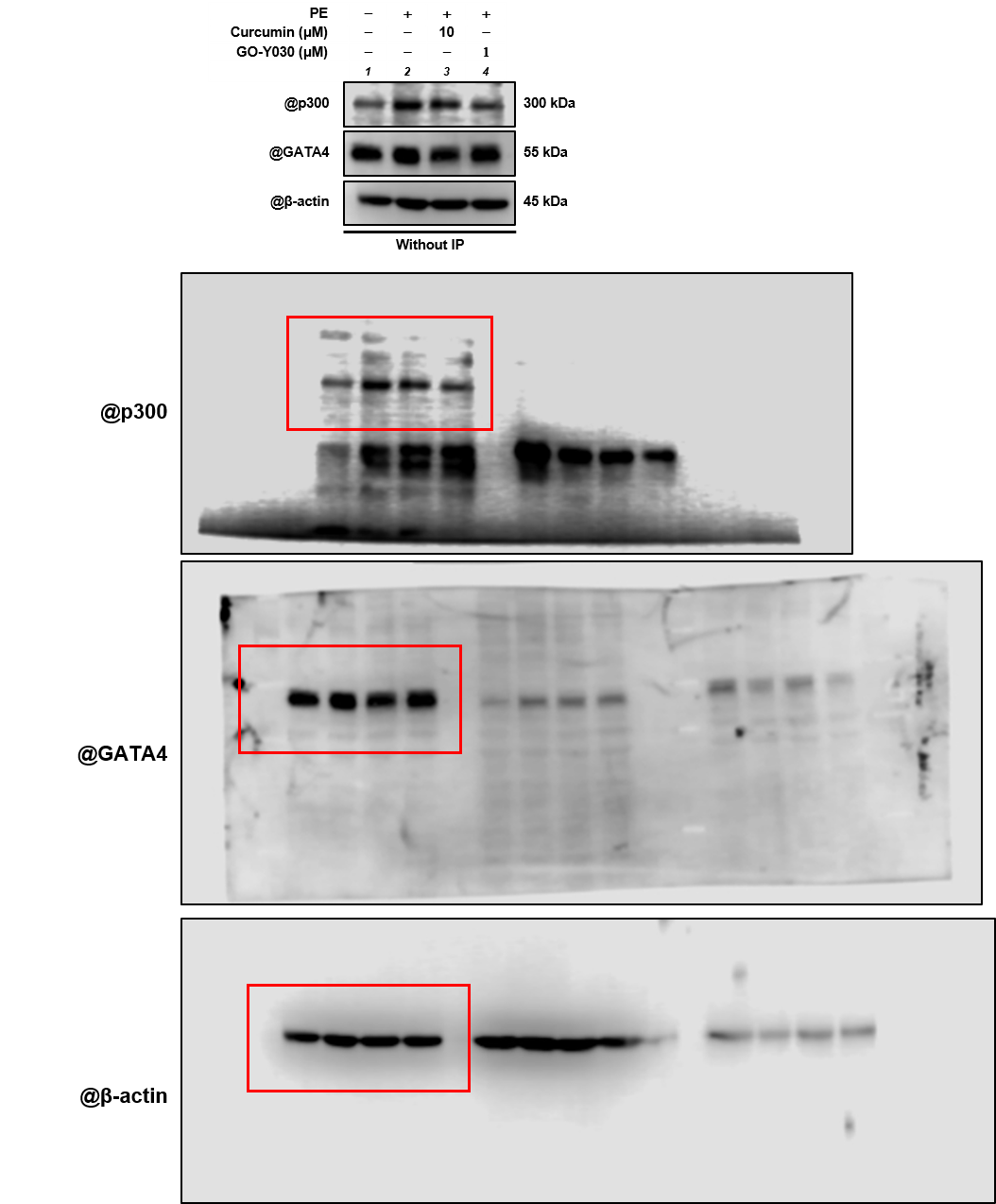


**Supplementary Fig. S7 Original western blotting images in Figure 3g**

Nuclear extracts prepared from primary cultured cardiomyocytes were subjected to western blotting with anti-p300 antibodies, anti-GATA4 antibodies, and anti-β-actin antibodies. The images in Figure 3g are shown in the red box.


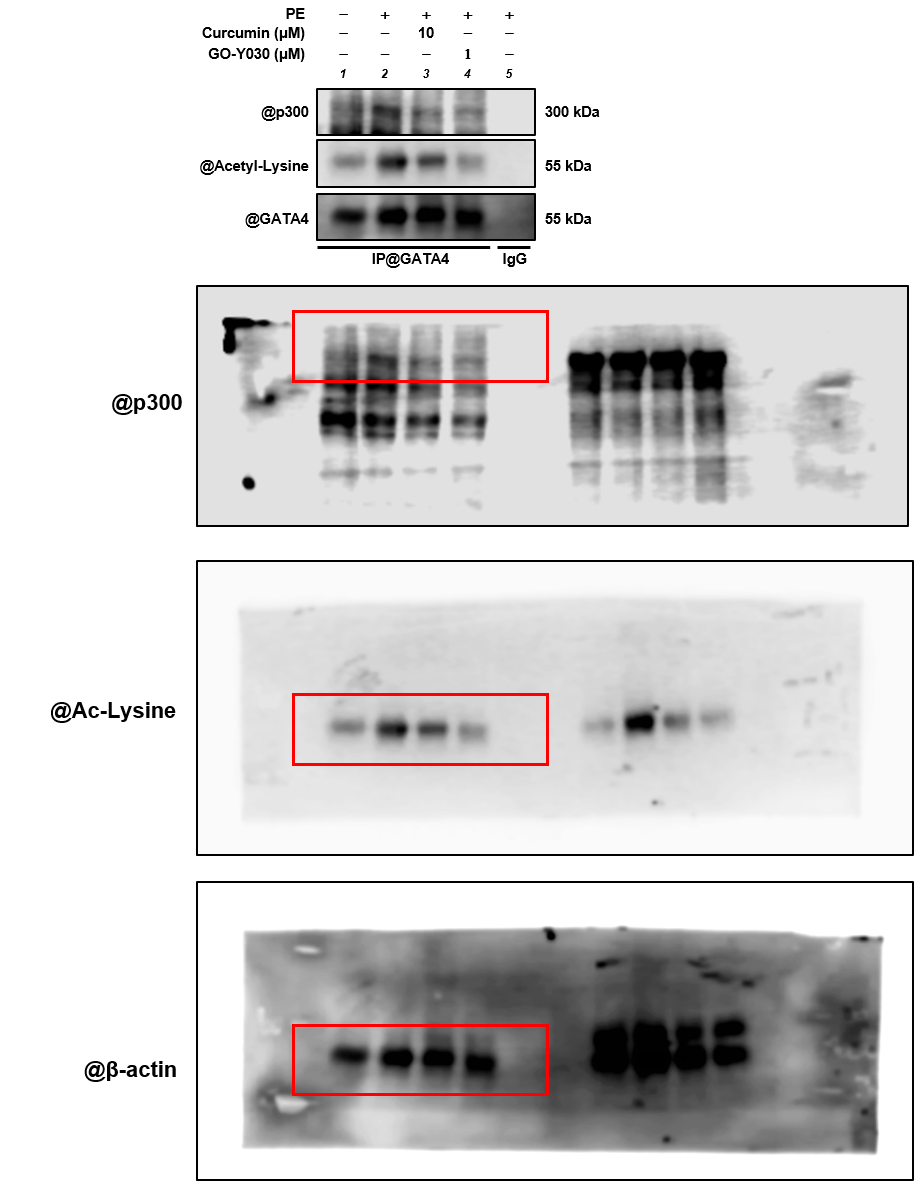


**Supplementary Fig. S8 Original western blotting images in Figure 3h**

The nuclear extracts were immunoprecipitated with goat anti-GATA4 polyclonal antibodies and subjected to western blotting with anti-p300 antibodies, anti-acetyl-lysine antibodies, and anti-GATA4 antibodies. The images in Figure 3h are shown in the red box.


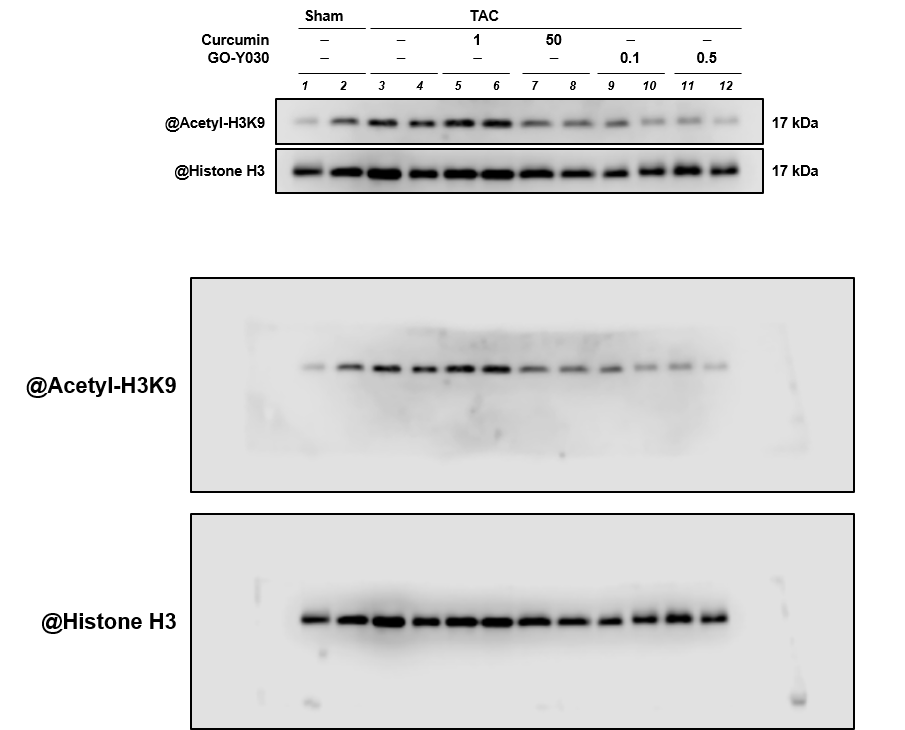


**Supplementary Fig. S9 Original western blotting images in Figure 7a**

Histone fractions from the mouse hearts were subjected to western blotting to assess acetylated histone H3K9 and total histone H3 levels.

**Supplementary materials.**

*Biochemical analysis*

Serum levels of blood urea nitrogen (BUN), creatinine (Cre), aspartate aminotransferase (AST), alanine aminotransferase (ALT), and total bilirubin (T-BIL) were measured using a biochemical blood analyzer (Hitachi 7180, Hitachi, Japan).

*Histological analysis*

Liver and kidney tissues were fixed with 10 % buffered formalin and embedded in paraffin. The sections were deparaffinized, and then the liver sections were stained with hematoxylin and eosin, and the kidney sections were stained with periodic acid-Schiff staining. The sections were then observed by microscope for hepatotoxicity and nephrotoxicity.

*Statistics*

Values are shown as the mean ± SEM from at least three independent experiments. Statistical comparisons were performed using ANOVA with the Tukey–Kramer test. A *p* value of < 0.05 was considered statistically significant.

| **Target gene** | | **Forward** | | | **Reverse** |
| --- | --- | --- | --- | --- | --- |
| **ANF** | **ATCACCAAGGGCTTCTTCCT** | | | **CCTCATCTTCTACCGGCATC** | |
| **BNP** | **TTCCGGATCCAGGAGAGACTT** | | | **CCTAAAACAACCTCAGCCCGT** | |
| **Collagen Type 1a1** | **AAGAAGACATCCCTGAAGTCA** | | | **TTGTGGCAGATACAGATCAAG** | |
| **Collagen Type 3a1** | **CCCAACCCAGAGATCCCATT** | | | **GAAGCACAGGAGGTGTAGA** | |
| **Fibronectin** | **CCGGTGGCTGTCAGTCAGA** | | | **CCGTTCCCACTGCTGATTTATC** | |
| **18S** | **CTTAGAGGGACAAGGGCG** | | **GGACATCTAAGGGCATCACA** | | |
| **Supplemental Table S1 Primer sequences**  ANF, BNP, and 18S primers have sequence homologies between rat and mice.  **Abbreviations**: ANF, atrial natriuretic factor; BNP, brain natriuretic peptide | | | | | |
